# Diet is Linked to Colorectal Cancer Risk among Asian Adults: A Scoping Review

**DOI:** 10.21315/mjms2023.30.3.2

**Published:** 2023-06-27

**Authors:** Lydiatul Shima Ashari, Ainaa Almardhiyah Abd Rashid, Shahril Mohd Razif, Lee Yeong Yeh, Hamid Jan Jan Mohamed

**Affiliations:** 1 Nutrition Programme, School of Health Sciences, Universiti Sains Malaysia, Kelantan, Malaysia; 2 Nutritional Science Programme and Centre for Healthy Ageing and Wellness (H-CARE), Faculty of Health Sciences, Universiti Kebangsaan Malaysia, Kuala Lumpur, Malaysia; 3 School of Medical Sciences, Universiti Sains Malaysia, Kelantan, Malaysia; 4 GI Function and Motility Unit, Hospital Universiti Sains Malaysia, Universiti Sains Malaysia, Kelantan, Malaysia

**Keywords:** colorectal neoplasms, diet, dietary exposure, Asia

## Abstract

This review aimed to map current evidence on the association between dietary factors and colorectal cancer (CRC) risk in Asia. This review was conducted based on Arksey and O’Malley methodological framework. Preferred Reporting Items for Systematic Reviews and Meta-Analyses extension for Scoping Reviews (PRISMA-ScR) flow diagram was used to record the review process. For the purpose of searching for articles, three electronic databases namely PubMed, EBSCOHost and ScienceDirect were employed. The inclusion criteria for articles selection were articles with association analysis between diet and CRC risk among Asians, had adults as participants, articles were written in English, open-accessed and published between years 2009 and 2021. Thus, 35 out of 369 screened articles were eventually included in this review which covered 28 case-control studies, six prospective cohort studies and one randomised clinical trial. Foods such as meats, alcohol and westernised diet have been shown to be associated with increase of CRC risk while fruits, vegetables and traditional meals decreased the risk of CRC. Only a few interventional and dietary patterns studies were identified. Specific single foods and nutrients and dietary patterns have been found to increase the risk but also protected the Asian population against CRC. The findings of this review will guide health professionals, researchers and policy makers to conduct a suitable study design and topic for future research.

## Introduction

Colorectal cancer (CRC) is currently a major public health concern worldwide due to the increasing trend of new cases from 1.36 million in 2015 (GLOBOCAN 2015) to 1.8 million in 2018 (GLOBOCAN 2018) (1, 2). CRC is the second leading cause of cancer-related mortality and the third most common cancer globally (1, 3). Despite having a lower overall frequency of CRC than Western countries, Asia has the highest number of prevalent instances, according to the International Agency for Research on Cancer’s analysis of the WHO database (4). The 5-year prevalence rate was reportedly higher in China, Japan, Korea, Malaysia, Singapore and Turkey than that of other Asian countries (≥ 46.5/100,000) while India, Indonesia, Vietnam and Iran had relatively low prevalence rate (5). The reason for these differences between Asian countries is not entirely clear. However, the large population size in China might be a contributing factor to its highest number of prevalent cases, new cases and deaths (4).

The root causes of CRC can be due to non-modifiable and environmental risk factors (4). Non-modifiable factors include genetic factors, age, gender, ethnicity, body height and family history of CRC while environmental risk factors consisted of smoking, alcohol drinking, overweight and obesity, westernised diet, physical inactivity, chronic diseases and microbiota (4). A genetic predisposition was estimated to cause 25% of CRC while 5% had reported to be inherited factors that contributed to its growth. Approximately 70% of CRC cases were sporadic cases and they were affected by environmental factors (6, 7). A systematic review and meta-analysis by Magalhães et al. (8) reported that a westernised diet characterised by a high intake of red and processed meat had increased the colon cancer risk. However, some foods might be protective factors against CRC risk such as whole grains, dairy products and foods containing dietary fibre based on a recent report from the continuous update project (CUP) (9).

Studies from various diet-related to CRC were conducted widely around the world (10–15). However, the literature mapping and research gaps especially in Asia are limited. Therefore, this review proposed to map the current evidence on the linkage between diet and CRC risk in Asia.

## Methods

This review was conducted following the methodological framework developed by Arksey and O’Malley (16) which included identifying the research question, identifying relevant studies, study selection, charting data and summarising the results. This review will be following the Preferred Reporting Items for Systematic reviews and Meta-Analyses extension for Scoping Reviews (PRISMA-ScR) (17). A protocol for the review does not exist and ethics approval was not required as the review relied solely on publicly available information.

### Identifying the Research Question

The review or research question was determined prior to identifying relevant studies. Population, Intervention, Comparison, Outcomes, Study type (PICOS) framework or model was applied to develop a review question and search strategy (18, 19). Three PICOS elements developed were: Asia (populations), diet (intervention or exposure) and CRC (outcomes) while another two elements (comparison and study type) were not applicable. The proposed review question was ‘What is the association between diet and CRC risk among Asians?’ Three key terms identified through the review question were diet, CRC and Asia. This is because the dietary habits of each country and region were different (20), Asia had the highest number of prevalent cases as compared to Western countries (4) and investigating the Asians habits may help to identify foods, nutrients and dietary patterns that causes CRC among the population.

### Identifying Relevant Studies

This review used three electronic databases namely PubMed, EBSCOHost and ScienceDirect for article searching. A simple search using the key terms: diet and CRC was performed in the electronic databases to determine the keywords (synonyms). Keywords were also obtained from Medical Subject Headings (MeSH) database. The key terms and keywords (Supplementary 1) obtained were then used in PubMed, EBSCOHost and ScienceDirect databases to search for the articles. The Boolean operator of AND was used to combine key terms and keywords (1 AND 2 AND 3) during the articles search. The number of studies available and selected based on the key terms and keywords search is shown in Supplementary 1.

### Study Selection

The article selections were conducted in two stages. For the first stage, two researchers were responsible to screen the titles and abstracts of the articles independently based on the inclusion and exclusion criteria and search terms. The inclusion criteria for the article’s search included articles that reported the association between diets and CRC risk among Asians, adults (aged 18 years old and above), all types of study designs except review articles, systematic reviews, narrative reviews, opinion papers, letters and conference proceedings with abstract only, the articles have to be written in English, open-accessed and published in between 1 January 2009 and 7 October 2021. Any non-human, animal studies and studies involving benign colorectal diseases were excluded. The PRISMA-ScR diagram was used as a guide to record the review process (17). After the selected titles and abstracts were screened and checked to ensure their relevance to the review question, researchers then retrieved the full text articles of selected abstracts and excluded unrelated abstracts.

In the second stage, the full articles were checked and reviewed by two researchers independently to ensure it meets the review objective. Furthermore, articles that do not reflect the association between dietary intake and CRC risk in Asia and articles that do not meet the review objective were excluded. Then, relevant articles were assessed to answer the review question. Discrepancies between researchers were discussed and a consensus was sought at the end. Throughout the process, EndNote® software was used to manage the results from the search.

### Charting the Data

A standardised data charting form (Table 1) was developed and reviewed by the study team for relevance and appropriateness. The form’s extraction fields captured relevant information on study characteristics, including author(s), year of publication, aims of the study, study design, type of scale or instrument, sample size, participant’s characteristics and results that were relevant to the objectives of the review. The charting form was pilot tested by researchers with a random sample of five sources of evidence to ensure all relevant data were captured. Eligible sources of evidence were charted using Microsoft Excel. The goal of this scoping review was to provide an overview of the existing literature regardless of quality and a formal appraisal of the methodological quality of sources of evidence that were included in the review was not performed.

### Summarising the Results

A numerical and descriptive summary of the charting results was used to present findings. Besides that, a summary diagram was used to better visualise the linkage between diet and CRC risk in Asia. The limitations of this study were also assembled to allow identification of research gap and produce useful recommendations for future research.

## Results

**Figure 1:**
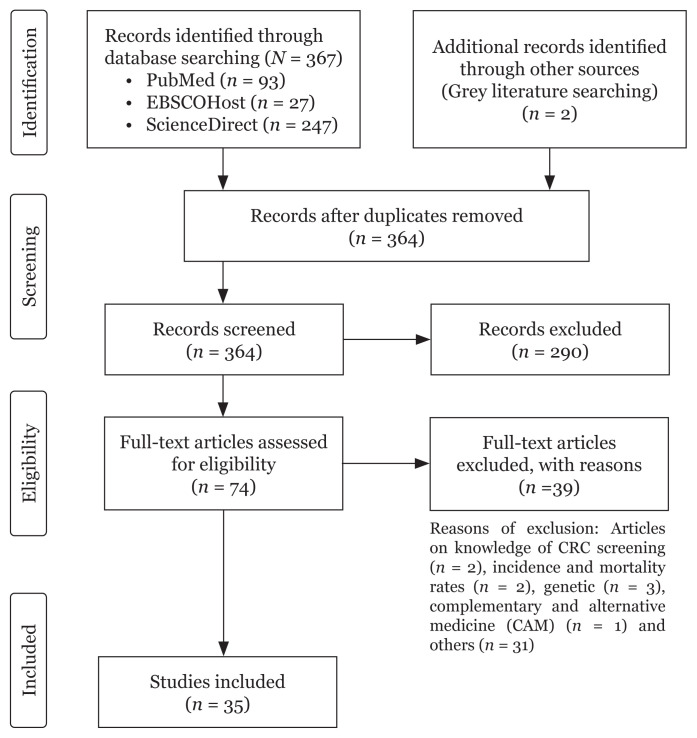
Process of articles selection based on PRISMA-ScR flow diagram

A total of 367 articles were identified from three search engines namely PubMed (*n* = 93), EBSCOHost (*n* = 27) and ScienceDirect (*n* = 247) while another two articles were obtained from other sources (Figure 1). Five duplicate articles were removed and the remaining 364 articles were screened based on the title and abstracts. Out of 364 articles, 290 were excluded because the titles and abstract did not match the research question of the study. The remaining 74 articles were assessed for eligibility based on their full text. A total of 39 articles were not eligible and excluded from the study as the articles did not analyse the association between CRC and diet. The final total articles included in this review were 35 and the outcome was presented in Table 1.

The included articles were carried out in 10 Asian countries which were China (*n* = 12), India (*n* = 2), Iran (*n* = 7), Japan (*n* = 6), Jordan (*n* = 4), Korea (*n* = 4), Oman (*n* = 1), Pakistan (*n* = 1), Taiwan (*n* = 2), Thailand (*n* = 4) and Vietnam (*n* = 2). These studies consisted of three study designs namely case-control (*n* = 28), prospective cohort (*n* = 6) and randomised clinical trial (*n* = 1) study.

**Figure 2:**
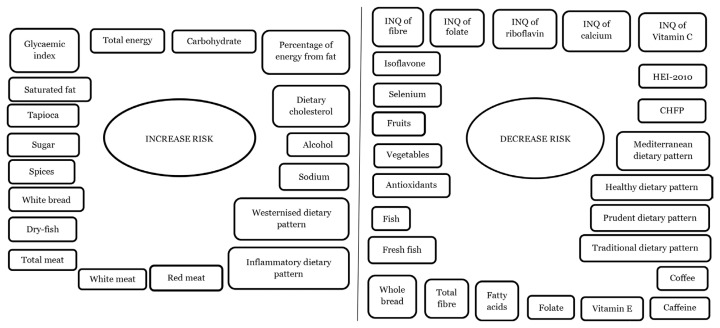
Single foods and nutrients and dietary patterns associated with increased and decreased risk of CRC Notes: INQ = index of nutritional quality; HEI-2010 = Healthy Eating Index-2010; CHFP = Chinese Food Pagoda

The results showed that various single foods and nutrients and dietary patterns (multiple food groups and multiple foods groups and nutrients) were related to the risk of CRC. A total of 24 primary studies indicated the association of CRC risk with single foods and nutrients while 11 studies, with dietary patterns. Single foods and nutrients and dietary patterns that increased risks were red, white and total meat, dry-fish, white bread, spices, sugar, tapioca, saturated fat, dietary glycemic index (GI), total energy, protein, carbohydrate, percentage of energy from fat, dietary cholesterol, alcohol, sodium, westernised diet and inflammatory factors. On the other hand, single foods and nutrients, and dietary patterns that decreased risks were fruits, vegetables, antioxidants, isoflavone, fish, fresh fish, whole bread, total fibre, fatty acids, index of nutritional quality (INQ) of fibre, folate, riboflavin, calcium, and vitamin C, folate, vitamin E, caffeine, coffee, selenium, traditional, prudent, healthy, Mediterranean, Chinese Food Pagoda (CHFP) and Healthy Eating Index-2010 (HEI-2010) (Figure 2).

## Discussion

The present review aims to systematically identify the existing literature on the relationship between diet and CRC risk in Asia. Based on the included literature (Table 1), total meat (21) and red meat (22, 23) including beef (24) and pork (25) are considered as single foods were associated with increase CRC risk in Asian countries. According to Hur et al. (26) the harmful substance known as heterocyclic amines (HCAs), polycyclic aromatic hydrocarbons (PAHs) and N-nitroso compounds (NOCs) that were typically produced during cooking and processing of red meat might induce cancer. Furthermore, Dao et al. (27) reported that heterocyclic amines 2-amino-1-methyl-6-phenylimidazo pyridine (PhIP) which was produced from cooked beef was associated with an increase in CRC risk. Besides red meat, white meat (23) including chicken (28) was found to contribute to CRC development. In addition, *Clostridium perfringens/histolyticum* spp. was found abundant in chicken meat than beef or salmon based on in vitro faecal batch culture investigation (29). It has been proclaimed to produce other enzymes involved in genotoxic or carcinogenic metabolites production in the colon such as β-glucosidase, nitroreductase and azoreductase (29).

Ganesh et al. (23) have observed that the consumption of dry-fish increased the risk of CRC among non-vegetarian Indian population. Dry-fish was considered as a risk factor probably due to the methods of preservation which consisted of salting and drying (23). Besides that, the high GI of white bread (grains) might explain why this diet was also associated with CRC risk among Jordanians (28). Moreover, Franceschi et al. (30) stated that refined carbohydrate was responsible for CRC based on their positive relationship with GI and glycaemic load (GL). Although the exact mechanism for cancer genesis with high GI type of dietary pattern is not clear, but insulin resistance or insulin growth factor might be involved (31). Spices such as chillies and pepper, sugar and tapioca were also associated with increased CRC risk (24). Chillies have carcinogenic properties due to their capsaicin compound and might explain the positive association between spices and CRC risk (24). Furthermore, the unprocessed tapioca contains toxins such as linamarin and cyanide derivatives. These toxins could directly act on the bowel mucosa to cause CRC (24).

Besides food items and food groups, macro- and micronutrients were also identified as contributors to the CRC risk in this current review. High fats diet (32), saturated fat (22, 33), dietary GI (34), total energy (33), protein (33), carbohydrate intakes (33), percentage of energy from fat (33), dietary cholesterol (33), alcohol (35) and sodium (33) intake were associated with increased risk of CRC where by, high intake of these nutrients among certain populations could be the reason why it leads to disease development.

However, there are certain food items and groups have been found to be protective against CRC. The consumption of fruits (24) and vegetables (24) including cabbage (23) and sprout (23) may reduce the development of CRC among the Indian population. A study by Xu et al. (36) reported that, antioxidants from fruits and vegetables such as flavan-3-ols, flavanones, flavones and flavonols were associated with decreased CRC risks among Chinese population. According to Khankari et al. (37), higher intake of isoflavone could reduce the CRC risk among premenopausal women with BMI *<* 23.0 kg/m^2^ at baseline assessment. Antioxidants trap the reactive oxygen molecules and free radicals at cellular level, thus intervening in a protective mechanism against oxidative damage (33). CRC risk could be reduced by approximately 25% among individuals who consume fish daily like the community in Kerala, India (24). They commonly consumed sea fish that were rich in vitamin D and selenium. According to Du and Fang (38), these nutrients may protect the pathogenesis and development of CRC. Another study conducted also in India showed that, fresh-fish intake had a 40% to 70% reduction in CRC development as compared to individuals who do not consume fresh-fish (23). Moreover, whole bread was classified as having a protective effect against CRC in a Jordanian (28) study and this is because whole grain products could be rich in antioxidants including trace minerals and phenolic compounds (39).

Macronutrients such as total fibre (34) were associated with a 53% reduction of CRC risk while the increased intake of polyunsaturated fatty acids (PUFA) (40) and linoleic acid (LA) (40) might decrease the risk in men and linolenic acid (ALA) (40) in women. For INQ of fibre, it was negatively associated with CRC risk (41). Micronutrients that could prevent the formation of CRC included folate (35, 63), vitamin E (33), caffeine (33), selenium (42) and INQ of folate, riboflavin, calcium and vitamin C (41). Folate influences the availability of methyl groups in the CRC pathway, which enhances deoxyribonucleic acid (DNA) synthesis and repair (35). Besides that, vitamin E and caffeine act as an antioxidant to fight against free radicals and effectively scavenge reactive oxygen molecules and hydroxyl radicals (33) while selenium which also act as an antioxidant protects against oxidative stress (42). On the other hand, Riboflavin (vitamin B2) plays an important role in DNA methylation, stability, synthesis and repair (43). For calcium, it can modulate the CRC-related cell signalling pathways and inhibit oxidative DNA damage while vitamin C, has antioxidative and pro-apoptosis effect, inhibit cell proliferation and reduce inflammation (43).

The CRC risk factors and protective factors were not due only to single foods and nutrients, but it was also influenced by dietary patterns. According to Park et al. (44), westernised dietary patterns characterised by high intake of meats, carbohydrates (fast foods), oil and sugar were associated with enhanced risk of CRC especially among Korean women. A positive association was also observed between the western patterns and CRC in Iran (45). Cho et al. (46) reported that higher consumption of C-reactive protein (CRP)-dietary pattern score (inflammatory diet) derived from reduced rank regression (RRR) was associated with increased CRC risk and a stronger risk was observed in rectal cancer than colon cancer. Inflammatory diet patterns not only could be obtained from RRR but they could also be derived from an index-based method such as dietary inflammatory index (DII). All studies included inflammatory diet patterns using DII (47–51) (*n* = 5) and showed a positive association with CRC risk. Another six dietary patterns including traditional (44), prudent (44), healthy (45), Mediterranean (52), CHFP (53) and HEI-2010 (52) dietary patterns were found to be protective against CRC.

This review found that findings of diet relationship with CRC between Asia and Western countries had similarities. The similarities could be seen in several studies conducted by Bradbury et al. (54), Sanjoaquin et al. (55), Castelló et al. (56) and Fung et al. (57). Bradbury et al. (54) indicated that red meat and alcohol enhanced the risk while fibre was found protective against CRC among United Kingdom (UK) population. Another study done in the UK population by Sanjoaquin et al. (55) revealed that white bread and alcohol use were associated with increased risk, but regular fruit eating was associated with decreased risk of CRC. According to Castelló et al. (56), high and low adherence to Mediterranean and westernised dietary patterns respectively may decreased CRC risk among the Spain population. According to Fung et al. (57), westernised eating habits were associated with colon cancer in United States (US) women. Even while the majority of foods were comparable, there were others such tapioca and dry fish, that were only available in Asia and had ties to the illness. Thus, investigating the food habits of the Asian population is essential.

There were several strengths and limitations of the current scoping review. Strength-wise, the current and updated evidence provided in this review are useful to develop dietary intervention programmes for the mitigation and prevention of CRC. Limitation-wise, first there was insufficient interventional study designs with the majority were observational studies (28 case-control and six prospective cohort studies) which are known to have bias. Secondly, only 11 studies were conducted on dietary patterns as compared to 24 studies that were on single foods and nutrients. Dietary patterns are preferred because this approach captures the overall diet which also includes variation of foods and combination of nutrients in a studied population. Thirdly, most of the included studies were not stratified based on the disease outcome or anatomical subsites such as proximal colon, distal colon and rectum.

## Conclusion

In conclusion, single foods and nutrients and dietary patterns such as meats, alcohol and westernised diet have been found to be associated with the increase risks of CRC while fruits, vegetables and traditional decrease the risks of CRC among Asian population. The limitations or gaps identified from this review will assist and guide health professionals, researchers and policymakers to design suitable study designs and topics related to diet and CRC for future studies.

## Supplementary Data

Supplementary 1 Search strategy using PubMed, EBSCOHost and ScienceDirect

**Table:** 

Search terms	No. of studies available	No. of studies selected ^a^
1. (“Colorectal cancer*” OR colorectal OR “colon cancer” OR colon OR “rectal cancer” OR rectum OR “colorectal neoplasms” OR “colorectal neoplasia” OR “colorectal carcinoma” OR “colorectal adenocarcinoma” OR “lynch syndrome” OR “hereditary nonpolyposis colorectal neoplasms”).	PubMed: 492, 736 EBSCOHost: 7,110 ScienceDirect: 172, 597	PubMed: 29 EBSCOHost: 3 ScienceDirect: 3
2. (Diet* OR “dietary pattern*” OR “dietary habits” OR “dietary factors” OR “dietary risk factors” OR “dietary intake” “dietary flavonoids” OR “dietary non-enzymatic antioxidant capacity” OR “diet quality” OR “inflammatory diet*” OR “inflammatory diet* pattern” OR nutri* OR “nutrient patterns” OR “eating patterns” OR food* OR meat OR poultry OR fish OR fiber OR whole-grain OR “whole grains” OR nut OR curcumin OR beverage* OR alcohol OR “green tea” OR “black tea” OR coffee OR milk OR dairy OR macronutrients OR micronutrient* OR calcium OR “vitamin D” OR iron OR “portion size*”).	PubMed: 3, 243, 871 EBSCOHost: 49, 468 ScienceDirect: 1, 337, 220	PubMed: 27 EBSCOHost: 2 ScienceDirect: 1
3. (Asia OR Malaysia OR China OR India OR Indonesia OR Pakistan OR Bangladesh OR Japan OR Philippines OR Vietnam OR Turkey OR Iran OR Thailand OR Myanmar OR South Korea OR Iraq OR Afghanistan OR Saudi Arabia OR Uzbekistan OR Yemen OR Nepal OR North Korea OR Sri Lanka OR Kazakhstan OR Syria OR Cambodia OR Jordan OR Azerbaijan OR United Arab Emirates OR Tajikistan OR Israel OR Laos OR Lebanon OR Kyrgyzstan OR Turkmenistan OR Singapore OR Oman OR Palestine OR Kuwait OR Georgia OR Mongolia OR Armenia OR Qatar OR Bahrain OR Timor-Leste OR Cyprus OR Bhutan OR Maldives OR Brunei).	PubMed: 1, 214, 094 EBSCOHost: 22,573 ScienceDirect: 1, 171, 376	PubMed: 19 EBSCOHost: 2 ScienceDirect:1

Notes:

a For ScienceDirect only these terms were used: “Colorectal cancer”, diet, “dietary patterns”, “dietary factors” and Asia

## Figures and Tables

**Table 1:** Characteristics of the studies included in the review

Author, year	Aim	Study design/Scale	Sample size	Participant characteristics	Results
Abu Mweis et al. (28), 2015	To examine the association between food groups (including grains, fruits, vegetables, milk, and meat and legumes) and CRC risk in Jordan	Case-control study/FFQ known as DHQ I	167 cases and 240 controls	CRC patients and hospital-derived controls, Jordan, percentage age groups < 50 yr. (44.8%), > 50 yr. (55.2%) for cases and < 50 yr. (53.2%), > 50 yr. (46.8%) for controls	There was a direct association between the risk of CRC and the frequency of consumption of chicken (OR = 2.52; 95% CI: 1.33, 4.77) Increased consumption of white bread was associated with increased CRC risk (OR = 3.13; 95% CI: 1.18, 9.25; *P*_trend_ = 0.042) Increased consumption of whole bread was associated with decreased CRC risk (OR = 0.32; 95% CI: 0.12, 0.84; *P*_trend_ = 0.042)
Arafa et al. (22), 2011	To evaluate the dietary pattern, sociodemographic and other lifestyle risk factors of CRC patients as compared to control subjects in Jordan	Case-control study/SQFFQ	220 cases and 220 controls	CRC patients and hospital-derived control, Jordan, mean age for cases and controls (56.3 ± 12.3 yr. for males and 53.7 ± 6.8 yr. for females), males (53.6 %), females (46.4%)	The frequency of consumption of fruits (*P* < 0.001) and vegetables (*P* < 0.001) was lower among CRC cases while the frequency of consumption of red meat and saturated fat (*P* < 0.001) was higher and positively associated with CRC risk
Cho et al. (46), 2018	To examine whether a specific dietary pattern reflecting inflammation was associated with a risk of CRC	Age and sex matched case-control study/SQFFQ-106 items	695 cases and 1,846 controls	CRC patients and hospital-derived controls, Korea, mean age 56.4 yr. (cases) and 56.1 yr. (controls)	High CRP-dietary pattern score was associated with increased risk of CRC (OR = 9.98; 95% CI: 6.81, 14.62, for highest vs. lowest quartile; *P*_trend_ < 0.001) The association of CRP-dietary pattern score was slightly stronger with rectal cancer (OR = 11.69; 95% CI: 6.81, 20.05 for highest vs. lowest quartile; *P*_trend_ < 0.001) compared to colon cancer (OR = 8.87; 95% CI: 5.55, 14.18 for highest vs. lowest quartile; *P*_trend_ < 0.001) The stronger association between CRP-dietary pattern score with rectal cancer than colon cancer was observed in women but not in men
Cho et al. (47), 2016	To examine the association between the DII and the risk of CRC through a case-control study conducted in Korea	Age-gender matched case-control study/SQFFQ-106 items	923 cases and 1,846 controls	CRC patients and hospital-derived controls, Korea, mean age 56.6 yr. (cases) and 56.1 yr. (controls)	A higher DII score was associated with an increased incidence of CRC (OR = 2.16; 95% CI: 1.71, 2.73 for highest vs. lowest tertile; *P*_trend_ < 0.001) A slightly weaker association was observed with proximal colon cancer (OR = 1.68; 95% CI: 1.08, 2.61 for highest vs. lowest tertile; *P*_trend_ = 0.02) A stronger association was observed among women (OR = 2.50; 95% CI: 1.64, 3.82 for highest vs. lowest tertile; *P*_trend_ < 0.001) compared to men (OR = 1.72; 95% CI: 1.30, 2.28 for highest vs. lowest tertile; *P*_trend_ < 0.001) Stronger associations were observed among subjects who were ≥ 50 yr. (OR = 2.61; 95% CI: 1.98, 3.43 for highest vs. lowest tertile; *P* for interaction = 0.004), engaged in physical activity (OR = 3.42; 95% CI: 2.37, 4.95 for highest vs. lowest tertile; *P* for interaction < 0.001) and did not smoke (OR = 2.58; 95% CI: 1.81, 3.68 for highest vs. lowest tertile; *P* for interaction = 0.03)
DellaValle et al. (58), 2014	To investigate the association between dietary nitrate and nitrite intake and risk of CRC in the Shanghai Women’s Health Study	Prospective cohort study/FFQ-77 items	73,188	Women, Shanghai, aged 40 yr.–70 yr.	Overall, nitrate intake was not associated with CRC risk (HR = 1.08; 95% CI: 0.73, 1.59; *P*_trend_ = 0.39) Among women with vitamin C intake below the median (83.9 mg/day) and hence higher potential exposure to NOCs, risk of CRC increased with increasing quintiles of nitrate intake (highest vs. lowest quintile HR = 2.45; 95% CI: 1.15, 5.18; *P*_trend_ = 0.02) No association among women with higher vitamin C intake (HR = 0.93; 95% CI: 0.44, 1.96; *P*_trend_ = 0.69) No association between nitrite intake and risk of CRC overall (HR = 1.05; 95% CI: 0.77, 1.42; *P*_trend_ = 0.78) or by low (HR = 1.10; 95% CI: 0.68, 1.79; *P*_trend_ = 0.74) or high (HR = 0.90; 95% CI: 0.55, 1.49; *P*_trend_ = 0.85) intake of vitamin C
Ganesh et al. (23), 2009	To determine the various factors associated with CRC such as tobacco, alcohol drinking and dietary items	Case-control study/Food items questionnaire	203 cases and 1,628 controls	CRC patients and hospital-derived controls, Mumbai, percentage age groups < 35 yr. (11.3%), 35 yr.–44 yr. (27.1%), 45 yr.–54 yr. (26.6%), 55 yr.–64 yr. (24.1%), 65+ yr. (10.8%) for cases and < 35 yr. (13.4%), 35 yr.–44 yr. (26.3%), 45 yr.–54 yr. (27.7%), 55 yr.–64 yr. (21.0%), 65+ yr. (11.5%) for controls	No significant excess risk for chewers (OR = 0.9; CI: 0.7, 1.4), (OR = 0.5; CI: 0.2, 1.1), (OR = 0.8; CI: 0.6, 1.2) among men, women and both sexes respectively compared to those without the habits No significant excess risk for alcohol drinkers (OR = 1.2; CI: 0.7, 2.1), (OR = 1.2; CI: 0.7, 2.2) among men and both sexes, respectively compared to those without the habits Cabbage-eaters among men (OR = 0.6; CI: 0.3, 0.9) and both sexes (OR = 0.5; CI: 0.3, 0.8) had 40% and 50% reduction in risk, respectively, compared to those who did not eat cabbage. No significant observed among women (OR = 0.5; CI: 0.2, 1.1) Sprout eaters among women (OR = 0.7; CI: 0.4, 1.2) and both sexes (OR = 0.5; CI: 0.4, 2.4) had 30% and 50% reduction in risk, respectively. No significant observed among men (OR = 0.5; CI: 0.3, 0.7) Men who consume dry-fish had an increase of 1.6-fold risk compared to those who did not eat dry-fish (OR = 1.6; CI: 1.0, 2.6) Fresh-fish eaters among both sexes (OR = 0.6; CI: 0.4, 0.9) and women (OR = 0.3; CI: 0.1, 0.5) had 40% and 70% reduction in risk compared to those who did not eat fresh-fish Meat-eaters among women (OR = 2.4; CI: 1.2, 4.7) had a 2.4-fold excess risk than non-meat-eaters. Meat-eaters among men also had excess risk (OR = 1.0; CI: 0.6, 1.7) than non-meat-eaters Dark-green-leafy-vegetables consumed among men (OR = 1.1; CI: 0.6, 2.0), women (OR = 1.3; CI: 0.6, 3.2) and both sexes (OR = 1.2; CI: 0.7, 1.9) did not have protective effect against CRC Root vegetables consumed among men (OR = 1.0; CI: 0.5, 2.0), women (OR = 1.9; CI: 0.7, 5.7) and both sexes (OR = 1.3; CI: 0.7, 2.2) did not show any protective effect against CRC Other vegetables eaten among men (OR = 1.8; CI: 0.5, 6.8), women (OR = 0.3; CI: 0.1, 1.1) and both sexes (OR = 1.0; CI: 0.4, 2.4) did not have protective effect against CRC
Huang et al. (34), 2018	To evaluate the association between dietary intake of total carbohydrate, non-fibre carbohydrate, total fibre, starch, GI and GL and CRC risk	Age-gender matched case–control study/FFQ-81 items	1,944 cases and 2,027 controls	CRC patients, hospital-derived controls and community-derived controls, Guangzhou, mean age 56.4 yr. (cases) and 56.2 yr. (controls)	Total fibre was related to a 53% reduction in CRC risk (aOR_quartile 4 v. 1_ 0.47; 95% CI: 0.39, 0.58; *P*_trend_ < 0.01) Dietary GI was positively associated with CRC risk (aOR _quartile 4 v. 1_ 3.10; 95% CI: 2.51, 3.85; *P*_trend_ < 0.01)
Khan et al. (32), 2015	To examine associations of dietary practices, addictive behaviour and bowel habits in developing CRC among patients in a low-resource setup	Age-gender matched case-control study/Structured questionnaire	74 cases and 148 controls	CRC patients, healthy individuals, Karachi, average age of cases 41.47 ± 15.48 yr	Individuals who consumed high fats diet had 98% higher chances to endure CRC compared to those who did not consume those diets (OR = 1.98; 95% CI: 1.13, 3.49; *P* = 0.017)
Kim et al. (35), 2012	To investigate the association between folate and alcohol intake, methylenetetrahydrofolate reductase (MTHFR) C677T polymorphism and CRC risk in Koreans	Case-control study/FFQ-dietary intake, structured questionnaire-alcohol	787 cases and 656 controls	CRC patients, hospital-derived controls, Seoul, age of cases between 30 yr. and 79 yr.	High folate intake was associated with reduced CRC risk (OR = 0.64; 95% CI: 0.49, 0.84; *P*_trend_ = 0.002 for high compared with low intake) High alcohol consumption was associated with increased risk of CRC (OR = 1.76; 95% CI: 1.26, 2.46; *P*_trend_ = 0.001 for high compared with low intake)
Mafiana et al. (59), 2018	To compare the lifestyle characteristics of cases and controls To determine if there was any difference in daily macronutrient intake between cases and controls To explore the sociodemographic and lifestyle indices that predicted CRC while controlling for potential confounding variables	Case-control study/FFQ-dietary intake, structured questionnaire-alcohol	109 cases and 170 controls	CRC patients, hospital-derived controls, Muscat, mean age 53.7 yr. (cases) and 57.4 yr. (control)	Alcohol consumption was not associated with CRC (OR = 0.91; 95% CI: 0.32, 2.58; *P* = 0.86)
Nayak et al. (24), 2009	To identify the dietary predispositions of the indigenous population of Malabar region of the state of Kerala, India	Age and sex matched case-control study/FFQ	108 cases and 324 controls	Adenocarcinoma of colon patients, hospital-derived controls, Kerala, mean age of the cases 55.6 ± 0.98 yr	A strong association was found between CRC and tapioca (OR = 2.7; *P* = 0.001), beef (OR = 4.25; *P <* 0.001) and pungent spices (OR = 9.62; *P* = 0.018) Fruits and vegetables showed a strong negative association (OR = 0.15; *P* = 0.002) Fish consumption on a daily basis showed a 25% reduction in risk Heavy consumption of sugar (OR = 2.80) showed significant high risk
Park et al. (44), 2016	To identify major dietary patterns among Koreans and to evaluate the associations of these patterns with CRC risk by gender, taking into account different anatomical subsites	Case-control study/SQFFQ-106 items	923 cases and 1,846 controls	CRC patients, hospital-derived controls, Korea, mean age of cases 56.6 ± 9.7 yr	Traditional and prudent patterns were inversely associated with CRC risk (OR = 0.35; 95% CI: 0.27, 0.46 for the highest intake tertile of pattern score vs. the lowest; *P*_trend_ < 0.001) and (OR = 0.37; 95% CI: 0.28, 0.48 for the highest intake tertile of pattern score vs. the lowest; *P*_trend_ < 0.001), respectively Westernised pattern showed a positive association especially among women (OR = 2.13; 95% CI: 1.35, 3.34 for the highest vs. lowest tertile; *P*_trend_ < 0.01) A decrease in CRC risk among those with the highest intake of the prudent pattern was observed in all anatomical subsites in both men (OR = 0.36; 95% CI: 0.19, 0.68; *P*_trend_ < 0.01 for proximal colon; OR = 0.21; 95% CI: 0.12, 0.36; *P*_trend_ < 0.001 for distal colon; OR = 0.28; 95% CI: 0.18, 0.44; *P*_trend_ < 0.001 for rectum) and women (OR = 0.28; 95% CI: 0.11, 0.71; *P*_trend_ < 0.01; OR = 0.27; 95% CI: 0.13, 0.54; *P*_trend_ < 0.001; OR = 0.45; 95% CI: 0.25, 0.83; *P*_trend_ = 0.01), respectively
Poomphakwaen et al. (25), 2014	To investigate the interaction between the presence of a polymorphism of the XRCC1 gene and known risk factors for colorectal cancer in Thailand	Age and sex matched case-control study/Semi quantitative food and beverage intake frequency questionnaire	230 cases and 230 controls	CRC patients, hospital-derived controls, Khon Kaen Province or neighbouring provinces, percentage age groups of cases < 45 yr. (23.9%), 45 yr.–55 yr. (27.8%), 56 yr.–65 yr. (31.3%), > 65 yr. (17%)	High frequency of pork consumption was significantly associated with an increased risk of CRC (OR = 1.49; 95% CI: 1.00, 2.21; *P* = 0.047) Fish (OR = 0.94; *P* = 0.862), fruit (OR = 0.91; *P* = 0.669), vegetables (OR = 0.94; *P* = 0.753), beef (OR = 1.20; *P* = 0.37), poultry (OR = 1.45; *P* = 0.06) and others were not associated with CRC
Rafiee et al. (50), 2019	To examine the relationship between E-DII and the risk of CRC and CAP in a case-control study in Iran	Age and sex matched case-control study/Semi-quantitative FFQ-148 items	134 CRC, 130 CAP, 240 controls	CRC patients, CAP patients, hospital-derived controls, Tehran, mean age 59 (49.25–64) yr. (CRC), 58 (51–64) yr. (CAP)	A significant positive association was observed between E-DII and CRC risk (aOR_continuous_ = 1.71; 95% CI: 1.41, 2.07) Subjects in the third (highest) tertile was five times more likely to have CRC compared to subjects in the first (lowest) tertile (OR_tertile 3 vs. 1_ = 5.08; 95% CI: 2.70, 9.56; *P*_trend_ < 0.0001)
Safari et al. (45), 2013	To identify dietary patterns and its association with the risk of CRC in Tehran, Iran	Age and sex matched case-control study/SQFFQ-125 items	71 cases and 142 controls	CRC patients and hospital-derived controls, Tehran, mean age 59.9 yr. (men) and 55.7 yr. (women)	Healthy dietary pattern was significantly associated with a decreased risk of CRC (OR = 0.227; 95% CI: 0.108, 0.478; *P <* 0.001) while an increased risk of CRC was observed with the Western dietary pattern (OR = 2.616; 95% CI: 1.361, 5.030; *P* = 0.004)
Shivappa et al. (48), 2017	To investigate the association between DII scores and CRC in a case-control study in a Jordanian population	Age, sex and occupation matched case-control study/FFQ	232 cases and 271 controls	CRC patients, hospital-derived controls, Jordan, mean age 53.8 yr.	Subjects with higher DII scores were at increased odds of CRC (OR_continuous_ = 1.45; 95% CI: 1.13, 1.85; *P* = 0.002; and OR_tertile 3 vs. tertile 1_ = 2.13; 95% CI: 1.23–3.72; *P*_trend_ = 0.007)
Shivappa et al. (49), 2018	To examine the association between DII scores and CRC in a case-control study conducted in Iran	Case-control study/FFQ-125 items	71 cases and 142 controls	CRC patients, hospital-derived controls, Tehran, mean age of cases 58.2 ± 10.5 yr.	Subjects with higher DII scores had a higher odds of CRC (OR _continuous_ = 2.20; 95% CI: 1.22, 3.96; *P* = 0.01; and OR _tertile 3 vs. tertile 1_ = 2.47; 95% CI: 1.10, 5.55; *P*_trend_ = 0.02)
Takachi et al. (21), 2011	To examine associations between the consumption of red and processed meat and the risk of subsite-specific CRC by gender in a large Japanese cohort	Prospective cohort study	80,658	Men and women, Japan, aged 45 yr.–74 yr.	Higher consumption of red meat was significantly associated with a higher risk of colon cancer among women (HR = 1.48; 95% CI: 1.01, 2.17; *P*_trend_ = 0.03) Higher consumption of total meat was significantly associated with a higher risk of colon cancer among men (HR = 1.44; 95% CI: 1.06, 1.98; *P*_trend_ = 0.07) Red meat was positively associated with proximal colon cancer in women (HR = 1.57; 95% CI: 0.95, 2.58; *P*_trend_ = 0.08) and distal colon cancer in men (HR = 1.42; 95% CI: 0.92, 2.19; *P*_trend_ = 0.12) No association between processed meat and risk of either colon (HR = 1.19; 95% CI: 0.82, 1.74; *P*_trend_ = 0.64) or rectal (HR = 0.98; 95% CI: 0.53, 1.79; *P*_trend_ = 1.00) cancer in women and colon (HR = 1.27; 95% CI: 0.95, 1.71; *P*_trend_ = 0.10) or rectal (HR = 0.70; 95% CI: 0.45, 1.09; *P*_trend_ = 0.25) cancer in men
Tayyem et al. (33), 2015	To investigate the association between macro- and micronutrient intake and CRC risk using data from a case-control study conducted in Jordan	Age, sex, occupation and marital status matched case-control study/FFQ	169 cases and 248 control	CRC patients, hospital-derived controls, Jordan, average age for cases 53.8 ± 12.2 yr.	Total energy intake was associated with a higher risk of developing CRC (OR = 2.60; 95% CI: 1.21, 5.56 for the highest vs. lowest quartile; *P*_trend_ = 0.03). Intakes of protein (OR = 3.62; 95% CI: 1.63, 8.05; *P*_trend_ = 0.002), carbohydrates (OR = 1.41; 95% CI: 0.67, 2.99; *P*_trend_ = 0.043) and percentage of energy from fat (OR = 2.10; 95% CI: 0.38,11.70; *P*_trend_ = 0.009) were significantly increased the CRC risk Saturated fat, dietary cholesterol and sodium intake were significantly associated with CRC risk (OR = 5.23; 95% CI: 2.33, 11.76; *P*_trend_ = 0.009; OR = 2.48; 95% CI: 1.18, 5.21; *P*_trend_ = 0.027; and OR = 3.42; 95% CI: 1.59, 7.38, respectively) Vitamin E and caffeine intake were protective factors against CRC risk (OR = 0.002; 95% CI: 0.0003, 0.011; *P*_trend_= 0.001) and (OR = 0.023; 95% CI: 0.008, 0.067) respectively
Wang et al. (60), 2014	To investigate the associations of sugars and sucrose intake with CRC risk in a community-based case–control study in Japan	Age and sex matched case-control study/Computer-assisted interview-148 items	816 cases and 815 controls	CRC patients, community-derived controls, Fukuoka and three adjacent areas, mean age 60.9 (8.8) yr. (men) and 60.0 (9.4) yr. (women)	Sugars (OR = 1.15; 95% CI: 0.75, 1.75; *P*_trend_ = 0.31; OR = 1.07; 95% CI: 0.64, 1.78; *P*_trend_ = 0.87) intakes were not related to CRC risk in men and women, respectively Sucrose (OR = 1.09; 95% CI: 0.71, 1.67; *P*_trend_ = 0.50; OR = 1.16; 95% CI: 0.69, 1.96; *P*_trend_ = 0.95) intakes were not related to CRC risk in men and women, respectively In men, sugars intake was associated with CRC risk inversely among never-smokers and positively among male ever-smokers (*P* for interaction = 0.01). Sugars intake was associated with an increased risk among men with no alcohol consumption but was unrelated to the risk among male alcohol drinkers (*P* for interaction = 0.02)
Xu et al. (36), 2016	To evaluate the associations between flavonoid intake from different dietary sources and CRC risk in a Chinese population	Age and sex matched case-control study/FFQ-81 items	1,632 cases and 1,632 controls	CRC patients, hospital-derived controls, healthy community controls, Guangzhou, mean age of cases 56.5 yr.	No significant association between total flavonoids and CRC risk (aOR = 1.06; 95% CI: 0.85, 1.32 for highest vs. lowest quartile, *P*_trend_ = 0.78) Flavanones and flavones intakes from total diet were negatively associated with CRC risk (aOR = 0.28; 95% CI: 0.22, 0.36 for highest vs. lowest quartile; *P*_trend_ < 0.01)
Abe et al. (61), 2016	To assess the association between GI, GL, and CRC using a prospective Japanese population-based cohort	Prospective cohort study/FFQ	73,501	Men and women, Japan, aged 40 yr.–69 yr.	Overall, no association was observed between GI and GL with CRC risk
Nakamura et al. (40), 2010	To investigate whether dietary instruction optimising the fat energy ratio suppresses the recurrence of colorectal tumours	Randomised clinical trial/3-day DR	373	Patients with two colorectal tumours (adenomas and/or early cancers) and has been removed endoscopically, Osaka, mean age 54.8 ± 6.1 yr. (men) and 56.3 ± 6.3 yr. (women)	In men, the risk of tumours decreased significantly as the intake of PUFA (OR = 0.48; 95% CI: 0.23, 1.02; *P*_trend_ = 0.04) and LA (OR = 0.42; 95% CI: 0.19, 0.89; *P*_trend_ = 0.02) increased In women, the risk of tumours decreased significantly as the intake of ALA (OR = 0.68; 95% CI: 0.18, 2.65; *P*_trend_ = 0.03) increased
Peterson et al. (62), 2009	To investigate whether coffee consumption was associated with decreased risk of CRC and whether cigarette smoking and stage of disease modify the association in the Singapore Chinese Health Study	Prospective cohort study/FFQ-165 items	61,321	Men and women, Singapore, mean age 56.5 yr.	No overall association between coffee intake and CRC In analysis by subsite and stage restricted to ever smokers, the coffee–colon cancer association between coffee and colon became significant for advanced disease (HR = 0.56; 95% CI: 0.35, 0.90; *P*_trend_= 0.01). Coffee may protect against smoking related advanced colon cancer
Panprathip et al. (63), 2019	To investigate the associations between folate status, gene polymorphisms and the risk of CRC in Thais and to identify the interactions of folate-gene polymorphism and their impact on CRC risk	Case-control study/SQFFQ-66 high-folate Thai food items	105 CRC, 101 CRA and 182 controls	CRC patients, CRA patients, hospital derived controls, Bangkok, percentage age groups < 50 yr. (10.5%), 50 yr.–59 yr. (23.8%), 60 yr.–69 yr. (31.4%), ≥ 70 yr. (34.3%) for CRC and < 50 yr. (4.0%), 50 yr.–59 yr. (25.7%), 60 yr.–69 yr. (26.7%), ≥ 70 yr. (43.6%) for CRA	The lowest quartile group of serum folate and folate intake had higher risk of CRC than the highest quartile group (OR = 11.45; 95% CI = 4.43, 29.59; *P* < 0.001) and (OR = 10.29; 95% CI = 4.17, 25.41; *P* < 0.001), respectively The risk of CRC was increased with alcohol consumption when combined with low folate status (*P* < 0.05)
Ting et al. (64), 2018	To identify factors associated with negative colonoscopy and positive iFOBT results obtained during CRC screening	Case-control study/-	648 colorectal neoplasia and 559 without colorectal neoplasia	Patients with colorectal neoplasia (positive colonoscopy group), patients without colorectal neoplasia (negative colonoscopy group), Taiwan, mean age of colorectal neoplasia patients 66.9 ± 10.6 yr.	Alcohol was not associated with colorectal neoplasia in positive iFOBT patients (aOR = 2.017; 95% CI: 0.840, 4.838; *P* = 0.116)
Wu et al. (65), 2009	To investigate the impact of betel quid chewing, cigarette smoking or alcohol consumption on CRC risk in Taiwan	Age and sex matched case-control study/Standardised questionnaire	258 CRC and 533 controls	CRC patients, hospital-derived controls, southern Taiwan, mean age of cases 63.1 ± 11.7 yr.	Alcohol drinking (aOR = 1.1; 95% CI: 0.7, 1.8; *P* for interaction = 0.608) and betel quid chewing (aOR = 1.3; 95% CI: 0.7, 2.4; *P* for interaction = 0.277) were not associated with CRC
Abulimiti et al. (51), 2020	To investigate whether the DII was associated with the risk of CRC in a Chinese population	Age and sex matched case-control study/FFQ-81 items	2,502 cases and 2,538 controls	CRC patients, hospital and community-derived controls, Guangzhou, mean age of cases 57.0 ± 10.3 yr.	A positive association was found between the E-DII and CRC risk (OR = 1.40; 95% CI: 1.16, 1.68; for highest vs. lowest quartile, *P*_trend_ < 0.01) Significant associations were not observed in women or underweight individuals when stratified based on cancer subsite, sex, BMI and smoking status
Bahrami et al. (41), 2020	To determine the relationship between the INQ and the risk of CRC and CRA	Age and sex matched case-control study/FFQ-148 items	129 CRC, 130 CRA and 240 controls	CRC and CRA patients, hospital-derived controls, Tehran, mean age of cases 56.6 ± 11.5 yr.	The INQ of calcium, riboflavin, vitamin C, fiber and folate were associated with decreased risk of CRC (ORcalcium = 0.21; 95% CI: 0.08, 0.52; *P* = 0.001; ORvitB2 = 0.35; 95% CI: 0.18, 0.65; *P* = 0.001; ORvitC = 0.16; 95% CI: 0.09, 0.28; *P* < 0.0001; ORfiber = 0.35; 95% CI: 0.21, 0.58; *P* < 0.0001; ORfolate = 0.33; 95% CI: 0.16, 0.65; *P* = 0.002, respectively)
Dao et al. (27), 2020	To examine the association between heterocyclic amines 2-amino-1-methyl-6-phenylimidazo pyridine (PhIP) and CRC in Vietnam	Case-control study/SQFFQ-97 items	512 CRC and 1,096 controls	CRC patients, hospital-derived controls, Hanoi	There was a significant association between PhIP intake and the risk of CRC among all participants (aOR = 4.89; 95% CI: 3.03, 7.89 for high intake vs. non-intake; *P*_trend_ < 0.01), men (aOR = 5.27; 95% CI: 2.83, 9.81 for high intake vs. non-intake; *P*_trend_ < 0.01) and women (aOR = 4.58; 95% CI: 2.10, 10.01 for high intake vs. non-intake; *P*_trend_ < 0.01) The significant positive association was observed for proximal colon (OR = 8.05; 95% CI: 3.86, 16.77; *P*_trend_ < 0.01 for total proximal colon; OR = 6.91; 95% CI: 2.65, 18.07; *P*_trend_ < 0.01 for men; and OR = 12.15; 95% CI: 3.73, 39.57; *P*_trend_ < 0.01 for women) The significant positive association was observed for distal colon (OR = 1.69; 95% CI: 0.49, 5.86; *P*_trend_ < 0.01 for total distal colon; and OR = 2.82; 95% CI: 0.75, 10.52; *P*_trend_ = 0.05 for men) The significant positive association was observed for rectum (OR = 4.24; 95% CI: 2.29, 7.84; *P*_trend_ < 0.01 for total rectum cancer; OR = 3.49, 95% CI: 1.48, 8.23; *P*_trend_ < 0.01 for men; and OR = 5.50; 95% CI: 2.10, 14.39; *P*_trend_ < 0.01 for women)
Jafari Nasab et al. (52), 2020	To investigate the association of HEI-2010 and MSDPS with CRC and CRA risk	Age and sex matched case-control study/FFQ-148 items	129 CRC, 130 CRA and 240 controls	CRC and CRA patients, hospital-derived controls, Tehran, median age 59 (49.25–64) yr. (CRC), 58 (51–64) yr. (CRA) and 56 (50–61.75) yr. (controls)	HEI-2010 (OR = 0.04; 95% CI: 0.01, 0.12 for highest vs. first tertiles, *P*_trend_ = 0.00) and MSDPS (OR = 0.19; 95% CI: 0.09, 0.38 for highest vs. first tertiles, *P*_trend_ = 0.00) were significantly associated with lower odds of CRC
Khankari et al. (37), 2020	To evaluate the association between soy isoflavones and soy protein and CRC risk using four prospective cohort studies from China and Japan	Prospective cohort studies/FFQ-11 and 138 items	205,060	Men and women, China and Japan, mean age 54.8 ± 8.9 yr.	No significant association between soy isoflavones (HR_isoflavones_ = 0.97; 95% CI: 0.86, 1.11; *P*_trend_ = 0.68) and soy protein (HR_soy protein_ = 0.98; 95% CI: 0.86, 1.12; *P*_trend_ = 0.67) with CRC risk in the pooled analysis No association among ever smokers consuming higher isoflavones (HR_isoflavones_ = 0.83; 95% CI: 0.68, 1.00; *P*_trend_ = 0.69) and soy protein (HR_soy protein_ = 0.81; 95% CI: 0.39, 1.10) Risk reductions were observed among premenopausal women with a BMI *<* 23.0 kg/m^2^ at baseline for higher isoflavone (HR_isoflavones_ = 0.58; 95% CI: 0.34, 0.98; *P*_trend_ = 0.65)
Kunnavuttivanich et al. (66), 2020	To explore the association between dietary patterns and disease recurrence among Thai CRC patients	Case-control study/FFQ-45 items	74 cases (recurrence), 151 controls (disease-free)	CRC patients (recurrence and disease-free), Thailand, percentage age groups < 65 yr. (56.8%), ≥ 65 yr. (43.2%) for cases and < 65 yr. (46.4%), ≥ 65 yr. (53.6%) for controls	Significantly low recurrence rates among patients consumed high amounts of pickled fish or chili-paste compared to patients who had never eaten those foods (*P* < 0.01) No significant association between meat/wheat (*P* = 0.58), fast-food/processed fruit (*P* = 0.34) or vegetarian (*P* = 0.85) dietary patterns and CRC recurrence
Luo et al. (42), 2021	To evaluate whether the association of dietary zinc and selenium intake and risk of CRC were modified by SOD1, SOD2, GPX and CAT polymorphisms in a Chinese population	Age and sex matched case-control study/FFQ-81 items	493 cases and 498 controls	CRC patients, hospital-derived controls and community-derived controls, Guangdong, mean age 57.8 yr. (cases) and 57.7 yr. (controls)	Intake of selenium was inversely associated with CRC risk (OR = 0.42; 95% CI: 0.28, 0.64; for highest vs. lowest quartile, *P*_trend_ < 0.001) Zinc was not associated with CRC risk. (OR = 0.96; 95% CI: 0.63, 1.47; *P*_trend_ = 0.505)
Nguyen et al. (53), 2020	To evaluate the association between CRC incidence and adherence to the CHFP, AHEI-2010 and DASH	Prospective cohort studies/SQFFQ-81 and 77 items	132,606	Men and women, Shanghai, median age for CRC 33.7 (6.2) and for non-CRC was 34.0 (6.0) yr.	CHFP score was inversely associated with risk of CRC (HR_Q2 vs Q1_ = 0.88; 95% CI: 0.77, 1.00; HR_Q3 vs Q1_ = 0.86; 95% CI: 0.75, 0.98; and HR_Q4 vs Q1_ = 0.84; 95% CI: 0.73, 0.96) with *P*_trend_ = 0.01 The inverse association appeared stronger for rectal cancer (*P*_trend_ = 0.01); age < 50 yr. (*P*_trend_ < 0.01); BMI < 25 kg/m^2^ (*P*_trend_ = 0.01) or without any metabolic conditions (*P*_trend_ < 0.01) at baseline, although no multiplicative interactions were noted The modified DASH (*P*_trend_ = 0.23) score and AHEI-2010 (*P*_trend_ = 0.27) showed no association with CRC risk

Notes: DII = dietary inflammatory index; GI = glycaemic index; GL = glycaemic load; CAP = colorectal adenomatous polyps; 3-day DR = 3-day diet record; SQFFQ = semi-quantitative food frequency questionnaire; CRC = colorectal cancer; CRA = colorectal adenoma; yr. = years old; E-DII = energy-adjusted DII; OR = odd ratio; aOR = adjusted odds ratio; DHQ I = diet history questionnaire I; LA = linoleic acid; PUFA = polyunsaturated fatty acids; ALA = linolenic acid; CI = confidence interval; CRP = C-reactive protein; iFOBT = immunochemical feacal occult blood test; INQ = index of nutritional quality; PhIP = heterocyclic amines 2-amino-1-methyl-6-phenylimidazo pyridine; HEI-2010 = Healthy Eating Index-2010; MSDPS = Mediterranean-Style Dietary Pattern Score; CHFP = Chinese Food Pagoda; AHEI-2010 = Alternative Healthy Eating Index-2010; DASH = Dietary Approaches to Stop Hypertension; vs. = versus; BMI = body mass index
